# Obstructive Shock Resulting From Right Atrial Collapse Compressed by Hepatomegaly: A Case Report

**DOI:** 10.7759/cureus.79905

**Published:** 2025-03-01

**Authors:** Yutaka Tsukamoto

**Affiliations:** 1 Internal Medicine, Médecins Sans Frontières, Cox's Bazar, BGD

**Keywords:** hepatomegaly, hypotension, obstructive shock, point-of-care ultrasound (pocus), right atrium collapse

## Abstract

A 57-year-old Rohingya man with chronic hepatitis C and a two-month history of abdominal swelling and bilateral leg edema presented to a hospital in a refugee camp. He was hypotensive (75/50 mmHg), and his hemoglobin was 5.3 g/dL. We found melena after admission. Hypotension persisted after intravenous fluid boluses followed by an infusion and transfusion. Point-of-care ultrasound revealed a significant collapse of the right atrium (RA), compressed by an enlarged liver, with a preserved left ventricular ejection fraction. Our assessment was obstructive shock due to RA collapse caused by hepatomegaly, in addition to hemorrhagic shock. Further extracellular fluid and transfusion improved his condition. RA collapse caused by hepatomegaly can cause obstructive shock.

## Introduction

Obstructive shock is usually caused by cardiac tamponade, pulmonary embolism, and/or tension pneumothorax [1,2]. The collapse of the right atrium (RA) is a characteristic finding in cardiac tamponade [3]. Also, patients with RA collapse can develop leg edema, and this can complicate the diagnosis of the cause of shock from physical examination because cardiogenic shock should be excluded. Several cases of RA compression without cardiac tamponade have been reported [4-7], but none of the patients developed shock. We successfully made a diagnosis with point-of-care ultrasound (POCUS) and treated a patient with shock and RA collapse that was probably caused by hepatomegaly by administering fluid and blood transfusions. We managed this patient in a hospital in a resource-limited refugee camp, where ultrasound machines are the only available diagnostic imaging modality. POCUS is useful for clinical decision-making in healthcare facilities that have insufficient diagnostic tests.

## Case presentation

A 57-year-old Rohingya man presented to a hospital in a refugee camp with a two-month history of abdominal swelling and bilateral leg edema. He denied any history of melena, hematemesis, fever, or dyspnea but described a history of chronic hepatitis C. He was not on regular medication. His vital signs included a blood pressure of 75/50 mmHg, a pulse rate of 114/min, a respiratory rate of 24/min, a percutaneous oxygen saturation (SpO_2_) of 96% on ambient air, a body temperature of 37.2°C, and a Glasgow Coma Scale score of E4V5M6. The physical examination revealed pale conjunctivae, abdominal distention without tenderness, and bilateral leg pitting edema.

The initial laboratory investigation showed a white blood cell (WBC) count at 11.5×10^9^/L, neutrophils at 7.3×10^9^/L (63.4%), lymphocytes at 3.5×10^9^/L (30.4%), hemoglobin at 5.3 g/dL, mean corpuscular volume at 82.8 fL, platelet count at 351×10^9^/L, alanine transaminase (ALT) at 28.1U/L, serum creatinine at 1.1 mg/dL, and random blood sugar at 89 mg/dL. The anti-hepatitis C antibody was positive. These results are shown in Table 1.

**Table 1:** Summary of laboratory investigations

Laboratory tests	Results	Reference value
White blood cells	11.5×10^9^/L	3.3-8.6×10^9^/L
Neutrophils	7.3×10^9^/L (63.4%)	40-60%
Lymphocytes	3.5×10^9^/L (30.4%)	26-40%
Hemoglobin	5.3 g/dL	13.7-16.8 g/dL
Mean corpuscular volume	82.8 fL	83.6-98.2 fL
Platelets	351×10^9^/L	158-348×10^9^/L
Alanine transaminase	28.1 U/L	10-42 U/L
Serum creatinine	1.1 mg/dL	0.65-1.07 mg/dL
Random blood sugar	89 mg/dL	73-109 mg/dL
Anti-hepatitis C antibody	Positive	Negative

We admitted the patient for fluid resuscitation and blood transfusion. After admission, he passed black stools. We made presumptive diagnoses of hemorrhagic shock, variceal bleeding, chronic hepatitis C, and liver cirrhosis. We administered two litres of Ringer's lactate solution and 450 mL of whole blood transfusion, followed by maintenance fluid. However, hypotension persisted at ~80/50 mmHg. Our concern was cardiac failure because of the patient's bilateral leg edema. We transfused another 450 mL of whole blood, started ceftriaxone 2 g/day to cover potential sepsis, including spontaneous bacterial peritonitis, and evaluated his volume status and cardiac function with POCUS.

**Figure 1:**
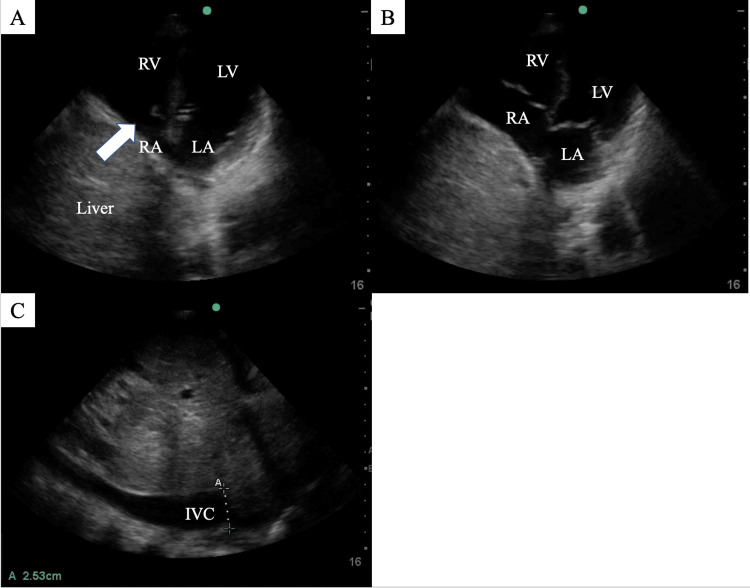
(A, B) Apical four-chamber view: The collapsed right atrium (RA) (a white arrow) compressed by the liver in the patient. (C) The patient’s dilated inferior vena cava (IVC). LA: left atrium; LV: left ventricle; RV: right ventricle

**Figure 2:**
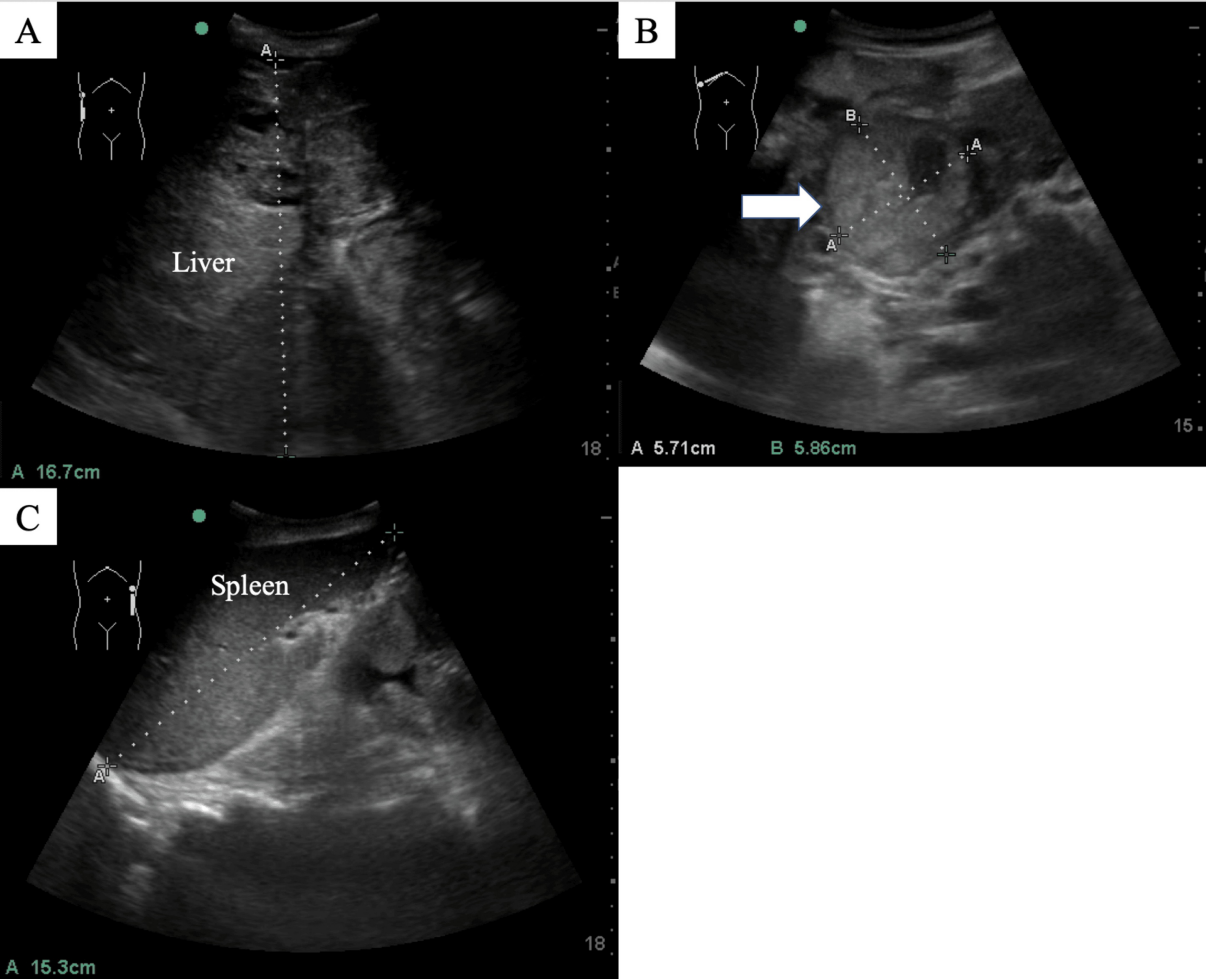
(A) The patient’s enlarged liver. (B) Liver mass suggestive of hepatocellular carcinoma (white arrow). (C) Splenomegaly.

The POCUS examination revealed a significant collapse of the patient's RA compressed by an enlarged liver (Figures 1-2 and Video 1). The craniocaudal length of the liver was 16.7 cm.

**Video 1:** Cardiac ultrasound (apical four-chamber view) showing a collapsed right atrium and preserved left ventricular systolic function.

The patient's left ventricular systolic function was preserved, and no pericardial effusion or right ventricle dilation was seen. The inferior vena cava (IVC) diameter was dilated at 25.3 mm, with reduced collapsibility (Video 2).

**Video 2:** Cardiac ultrasound showing a dilated inferior vena cava with reduced collapsibility.

Splenomegaly, ascites, and a 57.1 × 58.6 mm liver mass located between the left and right lobes, suggestive of hepatocellular carcinoma, were also identified (Figure 2).

Our assessment was obstructive shock due to RA collapse caused by hepatomegaly, in addition to hemorrhagic shock. Luckily, cardiac contractility was preserved. We thus administered additional extracellular fluid, and the patient's condition gradually improved. Blood pressure turned stable around 100/60 mmHg. We discharged him on day 9 of admission, and we advised him to visit a gastroenterologist for further evaluation, including variceal ligation, as this procedure is not available in our refugee-camp hospital.

## Discussion

We encountered a rare presentation of potential obstructive shock caused by a collapsed RA resulting from hepatomegaly. Obstructive shock is one of the causes of circulatory failure, and POCUS is extremely useful for the diagnosis of shock and the differentiation of the causes [1,2]. Our patient's hypotension persisted after resuscitation was provided with crystalloid solutions and blood transfusion, and he had bilateral leg edema, which suggested cardiac failure. POCUS was necessary to evaluate his cardiac function and volume status. Unfortunately, a chest X-ray was not available at our facility in a refugee camp. POCUS revealed the following findings: preserved left ventricular systolic function, collapsed RA compressed by an enlarged liver, and a dilated IVC. These findings are observed in obstructive shock. Our patient's melena was diagnostic of upper gastrointestinal tract bleeding, probably caused by a rupture of esophageal varices. Hypovolemic shock from bleeding was our initial assessment, but the patient's clinical response was not good, and the POCUS findings were compatible with obstructive shock, not hypovolemic or cardiogenic shock. We managed this obstructive shock with more fluid and blood transfusion, and the patient recovered. Generally, treatment of underlying causes is important in the treatment of obstructive shock, and in our case, a possible intervention would be a reduction of liver size, but it was impossible in our setting. We suppose that both hypovolemia and RA compression worsened the circulation.

Several cases of RA compression without cardiac tamponade due to various causes have been reported: diaphragm elevation [5,7], right hemidiaphragm eventration [6], and coronary artery aneurysm [4]. To the best of our knowledge, the present case report is the first describing RA collapse without cardiac tamponade and shock. A collapsed RA is one of the diagnostic findings in cardiac tamponade [3]. Our patient had hepatosplenomegaly, and this could have contributed to his RA compression, but because of our limited resources in a refugee camp, it was not possible to reach a confirmatory diagnostic testing by X-ray and computed tomography (CT). Right diaphragm elevation may have existed.

Imaging studies in resource-limited settings are not readily available. Our facility did not have X-ray. The availability of CT and magnetic resonance imaging (MRI) was much lower. On the other hand, ultrasound machines are getting cheaper and smaller and are more easily available all over the world, including developing countries. In addition, the diagnostic accuracy of handheld ultrasound devices is not inferior to comprehensive echocardiography [8,9]. POCUS is a powerful diagnostic tool in low-resource areas.

The key messages and novel findings of this case are as follows: (i) hepatomegaly could cause RA compression and obstructive shock; (ii) POCUS is beneficial for identifying underlying causes of shock, especially when suspected hypovolemic patients do not respond to fluid resuscitation; (iii) this condition could be managed by administering fluid and blood transfusions; and (iv) ultrasound is a useful diagnostic tool even in resource-limited settings.

## Conclusions

To the best of our knowledge, this is the first case report of shock and RA compression possibly related to hepatomegaly. POCUS is helpful when the cause of shock is not clear and the clinical response is not as expected. In this case, hypovolemic shock and cardiogenic shock were differential diagnoses because of melena and bilateral leg edema, but eventually, this patient might have both hypovolemic shock and obstructive shock. He responded well to infusion and transfusion and recovered dramatically. Ultrasound machines are widely available, even in resource-limited settings, and are effective in clinical decision-making.
